# Pulmonary Infection Caused by Mycobacterium terrae: A Case Report and Literature Review

**DOI:** 10.7759/cureus.6228

**Published:** 2019-11-25

**Authors:** Melanie Duran, Alan Araiza, Salim R Surani, Abhay Vakil, Joseph Varon

**Affiliations:** 1 Internal Medicine, Dorrington Medical Associates, Houston, USA; 2 Internal Medicine, Texas A&M Health Science Center, Temple, USA; 3 Internal Medicine, University of North Texas, Denton, USA; 4 Critical Care, United General Hospital, Houston, USA

**Keywords:** mycobacterium terrae, pulmonary infection, nontuberculous mycobacteria, atypical

## Abstract

*Mycobacterium terrae* infection can cause progressive debilitating disease. A case of a 63-year-old man with localized pulmonary infection characterized by extensive, thick-walled cavitary lesions is presented. A pneumonectomy was considered as definitive treatment, but the patient would not have tolerated the procedure given his severe deconditioning. Instead, he was placed on lifelong antibiotic treatment, but he continued to deteriorate and passed away. The slow-growing microorganism, *Mycobacterium terrae*, was isolated from bronchoalveolar lavage cultures seven weeks after specimen collection, five and a half weeks after the patient’s death. Clinical, microbiological and therapeutic data from this case and 16 other pulmonary cases from the literature are reviewed. Increased awareness of this microorganism will allow clinicians to consider *Mycobacterium terrae* in their differential diagnosis when dealing with nontuberculous mycobacteria infections.

## Introduction

*Mycobacterium terrae* was first isolated by Richmond and Cummings in 1950 from radish washings, which was originally simply called as the “radish bacillus”. This organism was described as a nonpathogenic saprophyte, as it did not cause any skin ulcerations or regional node involvement after being inoculated into guinea pigs [1]. Wayne, in 1966, described it as being abundantly found in soil, for which it received its name *M*. *terrae*, for the Latin *“Terra”* [2]. *Mycobacterium terrae* forms part of the *Mycobacterium* *terrae* complex, in conjunction with *M*. *nonchromogenicum* and *M*. *triviale*. These mycobacteria are described as slow-growing, nonchromogenic, acid-fast saprophytes, which exhibit high catalase activity, and β-galactosidase activity, which is not seen in other slow-growing mycobacteria [3].

Traditionally, *M*. *terrae* has been considered a contaminant. Despite being uncommon, there has been increasing evidence of infections caused by this microorganism. The most commonly affected areas are joints, tendon sheaths, bursae, and bones, with the tenosynovium of the hand and wrist being the most common site of infection [4]. Additionally, there are cases of infection involving lungs, skin, gastrointestinal tract, genitourinary tract, lymph nodes, or disseminated disease [5]. Here, we present a rare case of this challenging infectious disease affecting the lungs. 

## Case presentation

**Figure 1:**
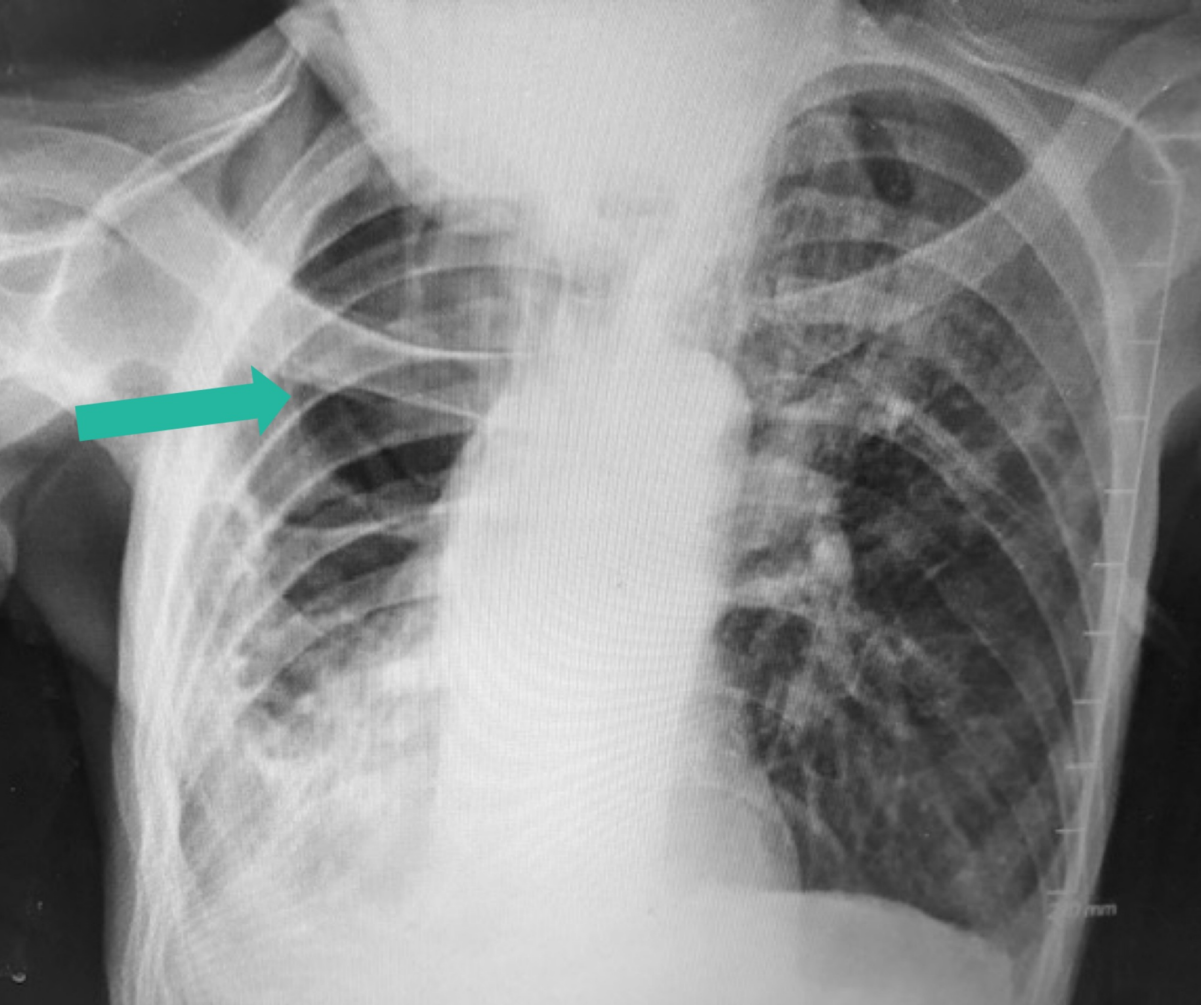
Chext X-ray on admission The patient’s chest X-ray showed left upper lobe and right basilar infiltration, with a cavitary process (green arrow) in right upper lobe.

A 63-year-old man with a history of chronic obstructive pulmonary disease, former one pack per day cigarette smoker for 10 years, degenerative joint disease, and seizure disorder initially presented to another facility in September 2015 with chronic productive cough and an abnormal chest X-ray. His physician there performed a bronchoscopy with bronchoalveolar lavage (BAL), which confirmed *M*. *terrae* infection. The patient was started on an unknown treatment regimen. Sixteen months later, in February 2017, he presented to the hospital with complaints of shortness of breath, productive cough with green sputum, chills, night sweats, generalized muscle weakness, loss of appetite, and cachexia with a body mass index of 11.8 kg/m^2^ and albumin of 2.6 g/dL. He denied fevers. The patient stated he was not eating and decided to go to the emergency department because he became so weak that he was unable to get up from bed. A chest X-ray was taken (Figure 1) which showed left upper lobe and right basilar infiltration, with a cavitary process in the right upper lobe.

**Figure 2:**
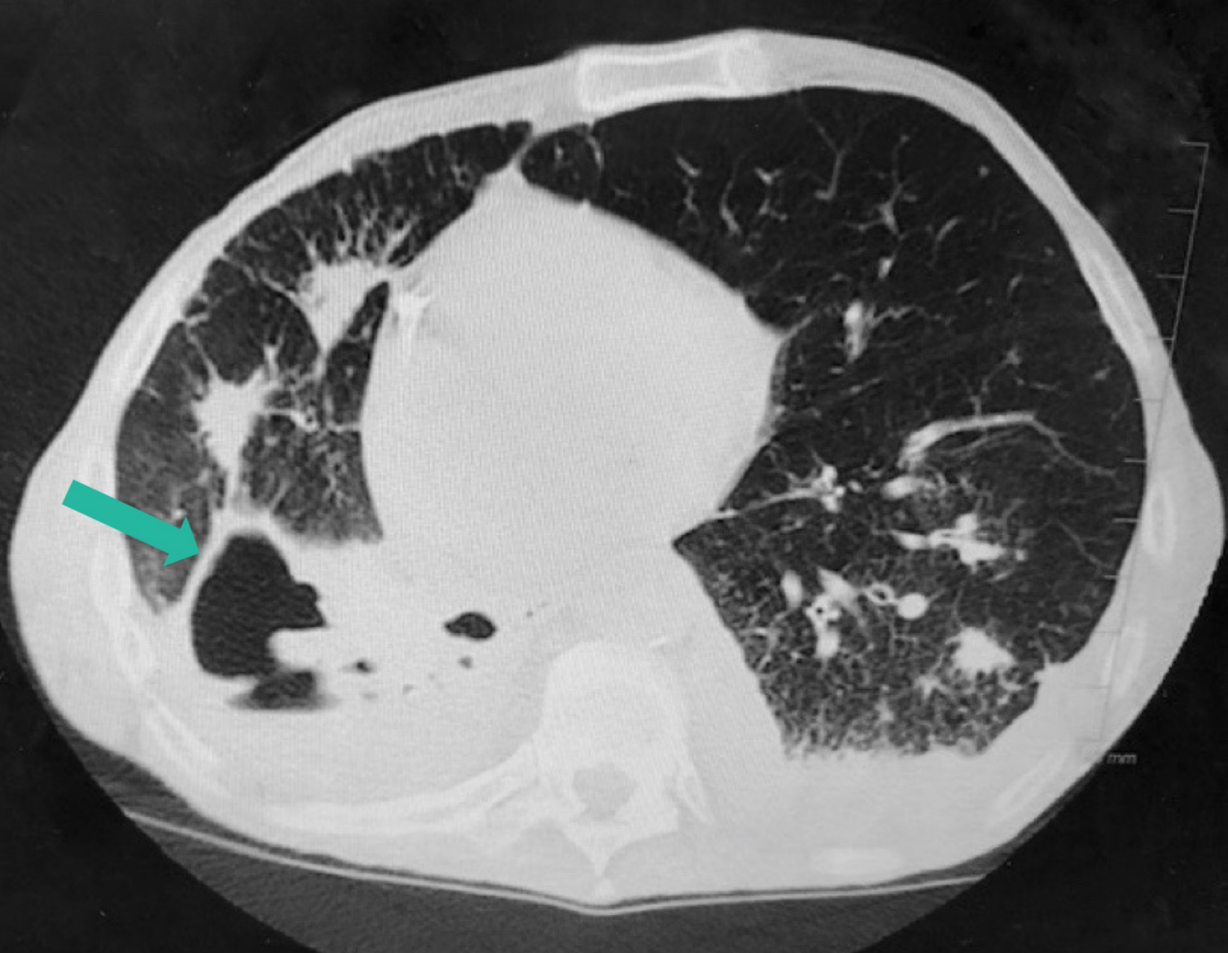
CT of the chest without contrast CT scan of the chest showed some chronic volume loss changes, bronchiectasis, and progression of the extensive thick-walled cavitary disease (green arrow) in the right lung with near complete replacement of the right lower lobe with thick-walled cyst and debris. Presence of consolidation, bronchiectasis, and multiple scattered pulmonary nodules in the left lung with interval enlargement in the lingular area.

**Figure 3:**
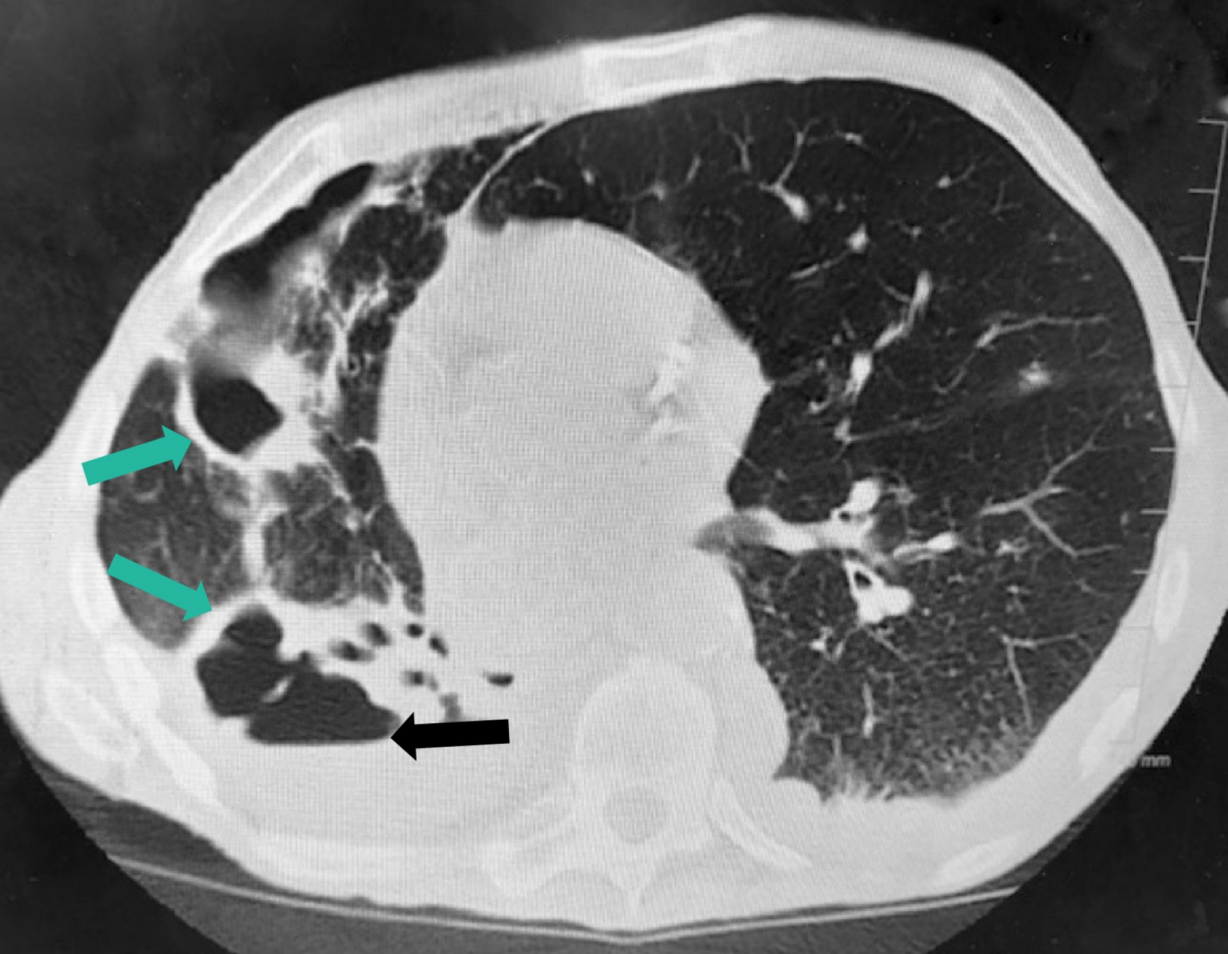
Second CT of the chest without contrast A large thick-walled cavity occupying a majority of the right hemithorax with increase in cavitary lesions (green arrows) in the right middle lobe and overall less aerated lung in the right hemithorax, new areas of parenchymal consolidation, cavitary and noncavitary nodules and patchy ground glass opacities in the left hemithorax with a new small left pleural effusion and a trace right pleural effusion are seen. There is an air-fluid level measuring 6.6 cm x 8.0 cm (black arrow). Findings overall suggest progression of extensive atypical infectious process.

A pulmonologist was consulted, and a computerized tomography (CT) of the chest showed a large cavitary lesion occupying the right hemithorax (Figure 2). Due to the previous diagnosis of *M*. *terrae* and worsening symptoms, a bronchoscopy was performed and he was started on an empirical antibiotic regimen of clarithromycin, ethambutol, and rifampicin. A repeat CT chest taken a week later showed progression of disease (Figure 3). A pneumonectomy was considered as an option, but the patient was deemed a poor candidate for the procedure given his severe protein calorie malnutrition, generalized weakness, and overall deconditioning. Therefore, he was placed on lifelong treatment with the three-medication regimen for the treatment of *M*. *terrae*.

Despite therapy, the patient continued to deteriorate and requested comfort measures. A hospice consult was placed, and a meeting with the family was arranged. His code status was do not resuscitate (DNR). He became hypoxic overnight and passed away the following morning. Bronchoalveolar cultures of the right lower lobe confirmed infection by *M*. *terrae* seven weeks after specimen collection, five and a half weeks after the patient’s death.

## Discussion

The prevalence and incidence of lung disease by *M*. *terrae* has been increasing worldwide since the first reported case of pulmonary infection in 1983 [5,6]. Case reports from around the world were reviewed, including pulmonary and extra pulmonary *M*. *terrae* infections, in order to gain better clinical recognition and reach an understanding of this pathogen's behavior [5]. Characteristics from 16 prior pulmonary cases and the one reported here are described in Table 1 [6-15].

**Table 1:** Reported cases of pulmonary infections by Mycobacterium terrae NS, not specified; TB, tuberculosis; DX, diagnosis; USA, United States of America; mo, months; y, years; M, male; F, female; H/O, history of; CF, cystic fibrosis; HIV/AIDS, human immunodeficiency virus/acquired immune deficiency syndrome; DJD, degenerative joint disease; Mt, Mycobacterium terrae; CXR, chest X-ray; CT, computerized tomography; ETH, ethambutol; RIF, rifampicin; INH, isoniazid; KM, kanamycin; SM; streptomycin; PEF, perfloxacin; PZA, pyrazinamide; CLAR, clarythromycin; CLIN, clindamycin; CIPR, ciprofloxacin; ETHO, ethoniamide; CYCL, cycloserine; RIBT, rifabutin.

First Author, Year	Case No	Country	Age, Sex	No. of Months Before Dx	Comorbid Conditions	Imaging Finding	Infected Site	Occupation	Basis for Dx	In Vitro Drug Susceptibility	Treatment	Treatment Duration	Outcome
Kuze, 1983 [6]	1	Japan	52, M	NS	None	CXR: cavitary lesion	Lung	Clerk	Sputum culture and resected tissue	ETH, ETHO	ETH, RIF, INH	11 mo; lobectomy at 5 mo	Cured
Tsukamura, 1983 [7]	2	Japan	57, M	NS	None	CXR: fibrocaseous lesion	Lung	Textile worker	Sputum culture	ETH, ETHO	KM, INH	NS	Died; cor pulmonale
Tsukamura, 1983 [7]	3	Japan	36, M	NS	None	CXR: cavitary lesion	Lung	Gilding worker (15 y)	Sputum culture	ETH	RIF, SM, INH	NS	Cured
Tsukamura, 1983 [7]	4	Japan	26, M	NS	None	CXR: cavitary lesion	Lung	Welder (8 y)	Sputum culture	ETH	RIF, SM, INH	NS	Cured
Tsukamura, 1983 [7]	5	Japan	74, M	NS	H/O TB	CXR: cavitary lesion	Lung	Clerk	Sputum culture	ETH	None	Never Init.	Died; cor pulmonale
Tsukamura, 1983 [7]	6	Japan	65, M	NS	H/O TB	CXR: bullous lesions	Lung	Salesman	Sputum culture	ETH	ETH, RIF, KM, INH	NS	Cured; cavity persisted
Tsukamura, 1983 [7]	7	Japan	59, M	NS	H/O TB	CXR: cavitary lesion	Lung	Public official	Sputum culture	ETH, ETHO	None	Never Init.	Died; cor pulmonale
Tsukamura, 1983 [7]	8	Japan	68, M	NS	H/O TB	CXR: cavitary lesion	Lung	Ex-railway worker	Sputum culture	ETH	ETH, RIF, KM	NS	Cured; cavity persisted
Krisher, 1988 [8]	9	USA	29, F	NS	None	CXR: Cavitary lesion	Lung	Bank teller	Sputum culture	ETH, RIF	RIF, ETH, INH	NS	Cured
Tonner, 1989 [9]	10	USA	64, M	NS	Alcoholism, smoker, subtotal gastrectomy	CXR: cavitary lesion w/ infiltrates	Lung	NS	Sputum culture	ETH, RIF, SM	ETH, RIF	18 mo	Cured
Palmero, 1989 [10]	11	Argentina	38, M	8 mo	Food allergy	CXR: cavitary lesion w/ patchyinfiltrates	Lung	NS	Sputum and gastric washing culture	RIF, CYCL	INH, RIF, PEF	NS	Cured
Peters, 1991 [11]	12	USA	64, F	NS	Ovarian carcinoma, bone marrow transplants x2	CXR: miliary infiltrates	Lung, skin	Housewife	Transbronchial biopsy	NS	ETH, SM, PZA	7 mo	Died; metastatic cancer
Spence, 1996 [12]	13	USA	61, F	NS	Smoker	CXR: large lung mass	Lung	NS	NS	NS	INH, RIF, ETH, PZA	NS	Cured
Carbonara, 2000 [13]	14	Italy	29, F	NS	HIV/AIDS	CT: miliary infiltrates	Lung, skin	NS	Sputum and urine culture	ETH, RIF, SM, RIBT, CIPR, CLAR	None	Never Init.	Died; resp. failure
Diaz Ricoma, 2001 [14]	15	Spain	50, M	NS	Smoker	CXR: cavitary lesion	Lung	Agriculturist	Sputum and BAL culture	ETH, RIF, SM	RIF, ETH, SM	14 mo	Cured
Lopez- Rodriguez, 2007 [15]	16	USA	13, F	6 mo	CF	CT: bilateral infiltrates	Lung	NS	Sputum culture	NS	ETH, CLAR	12 mo	Cured
Duran, 2019	17	USA	63, M	2 mo	Smoker, seizures, DJD, H/O Mt	CXR, CT: cavitary lesion	Lung	NS	BAL culture	Not performed	ETH, RIF, CLAR	10 days; died	Died

In the 17 cases analyzed, the age range of patients was 13 to 74 years, with a median age of 50 years. There were five females and 12 males. The time from the first clinical symptom to definitive diagnosis ranged from two to eight months, with a median of five months. The cases were reported from various countries including Japan (eight), United States (five), Spain (two), Argentina (one), and Italy (one) [6-15].

Of the 17 patients, 12 (71%) had an underlying chronic medical condition [7-15]. Of these, two patients (11%) were immunosuppressed: one with acquired immunodeficiency syndrome and the other with active malignancy receiving bone marrow transplant treatments [11,13]. There were nine patients with predisposing lung conditions including history of tuberculosis infection (four), chronic smoking (four), and cystic fibrosis with bronchiectasis (one) [7,9,12,14,15].

There was a predominant pattern of cavitary lesions noted in imaging studies of the chest in 11 (65%) patients [6-10,14]. Miliary infiltrates in the lungs were reported in the two (11%) immunosuppressed patients [11,13]. Other patterns of pulmonary infection reported in the remaining patients were fibrocaseous lesions (one), bullous lesions (two), large lung mass (one), and bilateral infiltrates (one) [7,12,15]. There was no correlation identified between occupation and exposure to the mycobacteria.

A review of treatment regimens used in these 17 patients showed that 14 (82%) patients were on multidrug regimens during their course of treatment. The mean duration for their treatment was 12 months. Three of the 17 patients did not receive any treatment at all. Two patients who did not receive treatment reportedly died of cor pulmonale, and one died of respiratory failure before he could initiate treatment [6,13]. Treatment duration was adjusted based on negative culture findings as well as improved clinical and radiographic evidence of an ongoing disease.

There was no single combined antibiotic regimen that yielded a 100% cure rate. There was a tendency toward combined therapies including ethambutol and rifampicin noticed in eight (47%) cases [6-9,12,14]. Of the 13 cases in which in vitro susceptibility tests were performed, 12 cases (92%) were susceptible to ethambutol, five cases (38%) to rifampicin, three cases (23%) to streptomycin, and another three cases (23%) to ethoniamide. Other nonresistant drugs included cycloserine (one), rifabutin (one), ciprofloxacin (one), and clarithromycin (one) [6-10,13,14]. Consistent with the literature reviewed, in vitro drug susceptibility to ethambutol, rifampicin, and a macrolide antibiotic makes this the most reasonable empirical regimen to initiate in suspected *M*. *terrae* infections.

Reported outcomes showed that eight patients (47%) were completely cured from their condition with combined antibiotic therapy alone, two (12%) were cured from infection but structural lung lesions remained, one (6%) was cured after a lobectomy and combined antibiotic therapy, and six (35%) cases resulted in death [6-15]. Statistical analysis regarding treatment and outcome relation cannot be performed due to too few cases reported. Our analysis was based on all of the reported cases of pulmonary infection by *M*. *terrae*. We acknowledge that there may be unreported cases by this pathogen that could change the result of the data reviewed. 

Patients who develop nontuberculous mycobacteria (NTM) lung disease usually have susceptibility factors, like structural lung damage, derived from conditions such as chronic obstructive pulmonary disease, bronchiectasis, prior history of tuberculosis, immunosuppression, primary ciliary dyskinesia, or cystic fibrosis, among others [16]. Classically, microorganisms of the *M*. *terrae* complex are considered contaminants and nonpathogenic colonies, rather than true causative agents of NTM lung disease. When found in cultures, however, their presence should be carefully interpreted [17]. After analyzing the literature, we found that this is the 17th case reported of pulmonary *M*. *terrae* infection worldwide.

In contrast with* M. tuberculosis*, isolation of *M*. *terrae* does not make the definitive diagnosis of NTM lung disease. According to the American Thoracic Society, criteria for diagnosis of pulmonary NTM disease, most recently updated in 2007, is based on integration of clinical, radiographic, and microbiological findings (Table 2) [18]. This case met all the criteria, fitting with the nonspecific symptomatology of NTM lung disease, an extensive thick-walled cavitary lesion on the right hemithorax on the chest CT, and a positive BAL culture for *M.* *terrae*.

**Table 2:** The 2007 ATS criteria for NTM lung disease ATS, American Thoracic Society; NTM, nontuberculous mycobacteria; HRCT, high-resolution computed tomography.

Clinical	Symptoms as: productive cough, hemoptysis, fatigue, malaise, weight loss, anorexia, dyspnea, and exclusion of other plausible diagnoses
Radiological	Fibrocavitary or nodular bronchiectatic aspect on imaging study or multifocal bronchiectasis with multiple small nodules on HRCT
Microbiological	Positive culture results from at least two separate sputum samples or positive culture from at least one bronchoalveolar lavage or washing or transbronchial, or other lung biopsy with mycobacterial histopathologic features (necrotizing granulomatous inflammation)

Isolation of acid-fast bacilli in a smear of sputum or BAL sample is usually the first sign that clinicians are dealing with a mycobacteria lung disease. Due to this, all the previously reported cases of pulmonary *M*. *terrae* infection were originally started on antituberculosis regimens, and after a slow three- to eight-week incubation period, microbiology services identified *M.* *terrae* and reported culture findings [6-15]. However, despite previous isolation in culture and diagnosis of pulmonary infection by *M*. *terrae*, there are no standard recommended treatment guidelines. The optimal antibiotic treatment is not established, but based on in vitro susceptibility, may include ethambutol, rifampicin, and a macrolide. After reviewing the literature, we found that the majority of cured cases followed a multi-antibiotic regimen for at least 12 months from the first negative culture [6,9,14,15]. In some cases, in which the microorganism persisted despite antibiotic therapy, amputation of the infected site, a partial or total pneumonectomy, was an alternative curative treatment [6]. In our analysis, we found that the antibiotics with the highest in vitro susceptibility were ethambutol, followed by rifampicin, and streptomycin. We acknowledge that this susceptibility is based on the limited data reviewed of the few reported cases. Testing in vitro susceptibility may be useful to measure the growth response of this isolate to specific drugs to cover the possibility of variants and adjust therapy accordingly. Significant in our case, the patient had prior history of *M. terrae* infection; therefore, a chronic *M*. *terrae* infection was highly suspected. A confirmatory bronchoscopy was performed, and the patient was started on an empiric antibiotic regimen of clarithromycin, ethambutol, and rifampicin.

In the literature, only one case reports a lobectomy as the definitive treatment for chronic pulmonary *M*. *terrae* infection not eradicated by antibiotic therapy [6]. We believe our patient could have benefited from a pneumonectomy of the affected area if he would not have been so deconditioned. Due to the severity of his disease, poor prognosis, and lack of standardized treatment guidelines, the patient was recommended lifelong antibiotic therapy, differing from prior cases where they only recommended therapy for 12 months to obtain complete eradication.

There have been reports hypothesizing that spontaneous resolution is part of the natural course of lung disease by *M*. *terrae* [11,14]. However, in the pulmonary cases reviewed, the absence of treatment correlated with death by respiratory insufficiency [7,13]. Our patient had a progressive course with fatal outcome, which raises concern of this inappropriately labeled “nonpathogenic” microorganism. Since the first pulmonary infection case by *M*. *terrae*, reported in 1983, there have been 17 cases reported [6-15]. Statistically, this is equivalent to one case reported every two years worldwide. For this reason, physicians should include this NTM in the differential diagnosis for atypical mycobacteria infections.

## Conclusions

*Mycobacterium terrae* should be considered a human pathogenic microorganism. Although initial isolation of *M. terrae* is usually considered a contaminant rather than a diagnosis, it should not be clinically disregarded right away, and further microbiological testing is recommended. Increased awareness amongst clinicians and microbiologists may improve the recognition of this NTM, which can have a fatal outcome, as it did with the patient presented.
